# Tight Junction Protein Claudin-12 Is Involved in Cell Migration during Metastasis

**DOI:** 10.3390/biom11050636

**Published:** 2021-04-25

**Authors:** Desislava Kolchakova, Dzhemal Moten, Tsvetelina Batsalova, Balik Dzhambazov

**Affiliations:** Department of Developmental Biology, Plovdiv University “Paisii Hilendarski”, 4000 Plovdiv, Bulgaria; kolchakova@uni-plovdiv.bg (D.K.); moten@uni-plovdiv.bg (D.M.); tsvetelina@uni-plovdiv.bg (T.B.)

**Keywords:** claudin-12, cell migration, antibody treatment, peptide inhibition, metastasis, tight junctions

## Abstract

Claudins are important components of the tight junctions determining barrier properties, cell polarity, and paracellular permeability. Although many functions of claudins in cancer cells have not been elucidated, recent studies have shown that claudins play an important role in cell migration and metastasis. Loss of epithelial/endothelial integrity, disruption of tight junctions, and increased paracellular leakage are often observed during metastasis. The aim of our study was to investigate the involvement of claudin-12 in the process of cell migration as well as to evaluate the possibility of using this protein as a specific target for the regulation of tumorigenesis. We have performed immunocytochemistry assays to detect the expression of claudin-12 in different epithelial/endothelial human cell lines, and selected three (A549, LS180, and HeLa) for further experiments. Using transwell chamber migration assays, we found that anti-claudin-12 antibodies inhibited both the migration and proliferation of claudin-12 expressing cells (A549 and LS180), inducing apoptosis, as well as the migration capacity of Jurkat cells through the monolayers formed from A549 or LS180 cells. In addition, co-cultures of Jurkat cells on monolayers from A549 or LS180 cells, in the presence of synthetic claudin-12 peptides representing the extracellular domains of the claudin-12 protein, also reduced the number of migrated Jurkat cells. Two of the tested peptides (p5 and p6) almost completely blocked the migration of Jurkat cells. All migrated Jurkat cells expressed LFA-1 and CD62L, but not CD44. Thus, claudin-12 is a suitable biomarker for tumor progression and metastasis and an attractive target for antitumor therapy. Anti-claudin-12 antibodies and competitive inhibitory peptides could be useful in the therapeutic approach applied to cancer metastasis in tissues expressing claudin-12.

## 1. Introduction

Claudins are small (20–34 kDa) tetraspanning transmembrane proteins that are involved in the structure of tight junctions [1,2]. They play a critical role in the regulation of paracellular permeability to ions and small molecules in endothelia or epithelia and the maintenance of cell polarity [3,4]. In addition, claudins are associated with various signaling pathways related to cell proliferation and differentiation [5,6].

The mammalian claudin gene family consists of 27 members, as their expression levels and subcellular localization depend on the tissue and cell type [7,8]. Among claudin proteins, claudin-12 is defined as an unusual member because it does not possess an intracellular PDZ binding motif [1,9], which mediates the interaction with the cytoskeleton. Claudin-12 is expressed in epithelia and endothelia of the gastrointestinal tract, inner ear, and brain endothelial cells [1,8,10], as well as in the smooth and striated muscle cells, neurons, and astrocytes [11]. It has been shown that in vitro expression of claudin-12 is up-regulated by vitamin D, suggesting an essential role for this claudin in Ca^2+^ absorption between intestinal epithelial cells [12].

Dysregulated expression of claudins has been reported in various cancers, suggesting that they may have an important role in the migration, invasion, and metastasis of cancer cells [3,13,14,15,16,17]. More than 90% of cancer-related deaths are due to metastases [18]. The metastatic process requires transendothelial migration of the cancer cells. They have to leave their primary site by intravasation in the lumen of the vasculature, to circulate in the bloodstream, and to extravasate to a secondary site [18,19]. Metastasis is accompanied by disruption of tight junctions, loss of epithelial/endothelial integrity, and increased paracellular leakage, providing a space for the mobility of the cancer cells [20]. It was reported that overexpression of claudin-12 significantly increased the metastatic properties of human bronchial epithelial cells BEAS-2B [17].

However, the role of claudins in migration of cancer cells through the tight junctions during metastasis is not fully understood. The aim of this study was to investigate the involvement of tight junction protein claudin-12 in the process of metastasis as well as to evaluate the possibility of using this protein as a specific target for the regulation of tumorigenesis. We hypothesized that cancer cells use claudin-12 to migrate through the tight junctions during metastasis and that blocking this protein or the competitive binding of cancer cells to peptides derived from the extracellular part of claudin-12 will reduce the metastatic process.

## 2. Materials and Methods

### 2.1. Cell Lines and Culture Conditions

The human cell lines A549 (epithelial lung carcinoma cells, ATCC CCL 185, NBIMCC 2404), Caco-2 (epithelial colon adenocarcinoma cells, ECACC 86010202), HT-29 (epithelial colon adenocarcinoma cells, ECACC 91072201), LS180 (Dukes’ type B epithelial colon adenocarcinoma, mucin-secreting cells, ECACC 87021202), SK-Hep-1 (endothelial liver adenocarcinoma cells, ATCC HTB 52, NBIMCC 1858), HeLa (epithelial cervix adenocarcinoma cells, ATCC CCL 2, NBIMCC 164) and Jurkat E6.1 (acute lymphoblastic leukemia T cells, ECACC 88042803) were obtained either from the European Collection of Authenticated Cell Cultures (ECACC, Salisbury, United Kingdom) or from the National Bank for Industrial Microorganisms and Cell Cultures (NBIMCC). All adherent cell lines were cultured in complete medium (CM) containing Dulbecco’s modified Eagle’s medium (DMEM) supplemented with 10% heat-inactivated fetal bovine serum (FBS) and stabilized antibiotic antimycotic solution (all from Merck KGaA, Darmstadt, Germany) at 37 °C, 5% CO_2_, 95% atmospheric air in a humidified incubator. The Jurkat cells were maintained at the same conditions using RPMI 1640 medium (Merck KGaA, Darmstadt, Germany) instead of DMEM. Prior to the performed assays, the cell lines were expanded in 75 cm^2^ culture flasks (TPP, Trasadingen, Switzerland).

### 2.2. Reagents and Antibodies

**Figure 1:**
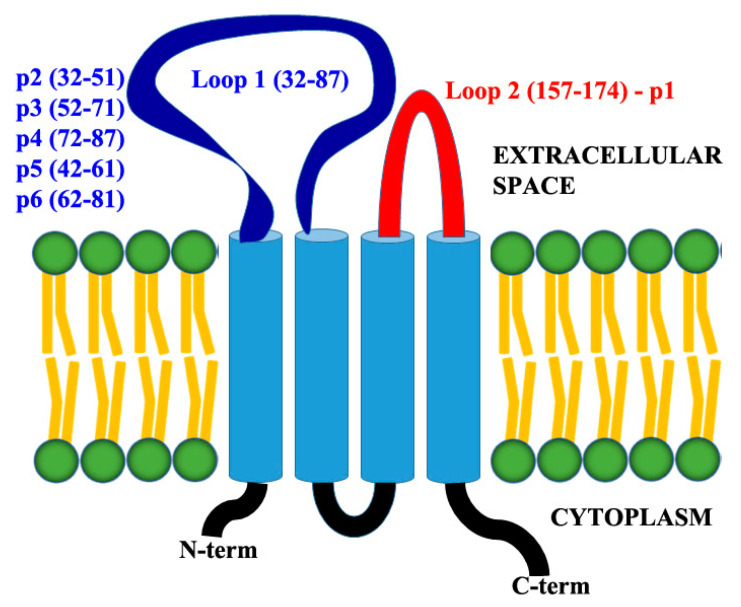
Model of claudin-12 protein showing the extracellular domains (loop-1 and loop-2) and designed peptides. Peptide 1 (p1) represents the sequence of loop-2/red, CLDN12 (157–174). Peptides p2, p3, p4, p5, and p6 represent the sequence of loop-1/blue, CLDN12 (32–87).

All reagents were of analytical quality and obtained from Merck KGaA (Darmstadt, Germany), unless otherwise stated. Six synthetic peptides (p1-peptide, YNIHLNKKFEPVFSFDYA, Cldn-12 (157-174), second extracellular loop; p2-peptide, NWRKLRLITFNRNEKNLTVY Cldn-12 (32-51); p3-peptide, TGLWVKCARYDGSSDCLMYD Cldn-12 (52-71); p4-peptide, TTWYSSVDQLDLRVLQ Cldn-12 (72–87); p5-peptide, NRNEKNLTVYTGLWVKCARY Cldn-12 (42–61); and p6-peptide, DGSSDCLMYDTTWYSSVDQL Cldn-12 (62-81), parts of the first extracellular loop) representing sequences from the extracellular domains of the protein claudin-12 (Figure 1) were synthesized from Schafer-N (Copenhagen, Denmark). Monoclonal anti-CLDN12 antibody produced in mouse (clone 2D8, cat. No SAB1403012), anti-mouse IgG (whole molecule)–FITC antibody produced in goat (cat. No F0257), and anti-mouse IgG (whole molecule)–Peroxidase antibody produced in goat (cat. No A4416) were purchased from Merck KGaA (Darmstadt, Germany). Monoclonal anti-human CD11a/CD18-APC (LFA-1) antibody (clone m24, cat. No 363410), anti-human CD62L-FITC (L-selectin) antibody (clone DREG-56, cat. No 304804), and anti-human CD44-PE antibody (clone BJ18, cat. No 338808), all produced in mouse, were purchased from BioLegend^®^ (San Diego, CA, USA). Annexin V-FITC and propidium iodide (PI) were purchased from Abcam (Cambridge, UK).

### 2.3. Immunocytochemistry and Western Blotting

A549, Caco-2, HT-29, LS180, SK-Hep-1 and HeLa cells were seeded (1.0 × 10^5^ cells/well) on coverslips in 12-well plates (TPP, Trasadingen, Switzerland) and cultured in a humidified incubator at 37 °C, 5% CO_2_ in complete DMEM (1 mL/well). After 48 h incubation period, the cells were washed twice with Dulbecco’s phosphate buffered saline (DPBS) and fixed with ice cold acetone:methanol (1:1). Then, cells were stained for 30 min with anti-CLDN12 antibody at room temperature, washed with DPBS, and incubated for 15 min with secondary anti-mouse IgG–FITC antibodies in darkness. After washing 3 times with DPBS, the expression of claudin-12 in the studied cell lines was observed using a fluorescent microscope (Leica Microsystems GmbH, Wetzlar, Germany). For the next experiments, we selected two claudin-12 positive cell lines (A549 and LS180) and one negative (HeLa).

For Western blot analysis of claudin-12, cells (A549, LS180, and HeLa) were washed twice in cold DPBS and lysed with RIPA lysis buffer (150 mM NaCl, 0.5 mM EDTA, 1% Triton X-100, 0.1% sodium dodecyl sulfate, 50 mM Tris-HCl, pH 8.0) containing a cOmplete™ protease inhibitor cocktail (Merck KGaA, Darmstadt, Germany). Lysates were sonicated for 20 s and centrifuged at 6000× *g* for 20 min. The supernatants were then filtered and concentrated by using 30 kDa and 10 kDa Amicon^®^ Ultra-15 Centrifugal Filter Units (Millipore). Protein concentrations were measured on a NanoDrop 2000 UV-Vis spectrophotometer (Thermo Fisher Scientific, Wilmington, DE, USA). Equal amounts (30 μg) of proteins (between 10–30 kDa) from the 3 cell lines were subjected to non-heat-denatured protein gel electrophoresis (4–20% polyacrylamide gradient ready mini-gels, Bio-Rad Laboratories, Hercules, CA). Separated fractions were electrotransferred onto a nitrocellulose membrane followed by blocking with 5% nonfat dry milk in DPBS for 1 h, then blotted with anti-CLDN12 antibodies at 4 °C overnight. After washing, blots were incubated with peroxidase-conjugated goat anti-mouse IgG (1:2000 dilution) at room temperature for 1 h. Immunoblots were developed using Pierce™ ECL Western Blotting Substrate (Thermo Fisher Scientific, Rockford, IL, USA) according to the manufacturer’s instructions.

### 2.4. Cell Migration Assays

For the cell migration assays of the selected adherent cell lines (A549, LS180, and HeLa), 5.0 × 10^5^ cells/well suspended in 100 μL complete medium (CM) were added in triplicates into the upper chambers of Corning^®^ HTS Transwell^®^ 96 well permeable supports (8.0 μm pore polycarbonate membrane, cat. No CLS3374, Merck KgaA, Darmstadt, Germany) and incubated at 37 °C in a humidified incubator containing 5% CO_2_ for 24 h. After 24 h of incubation, cell monolayers in the upper chambers were treated with monoclonal anti-CLDN12 antibody (1 μg/mL) for 30 min at 37 °C, then washed 3 times with 150 μL serum-free DMEM and incubated for an additional 24 h in 100 μL serum-free DMEM at 37 °C in a humidified atmosphere containing 5% CO_2_. For the last 24 h of incubation, 100 μL of complete medium was added to the lower chambers. Cells without anti-CLDN12 antibody treatment served as control groups. Cells migrated into the lower chambers were fixed in cold methanol and stained with 0.5% crystal violet for 10 min. All migrated cells were visualized under an Inverso inverted light microscope (Medline Scientific, Chalgrove, Oxfordshire, UK) equipped with a Si-3000 digital camera and software (Medline Scientific, Chalgrove, Oxfordshire, UK). The cells were counted in each transwell of the triplicates and photographed (magnification, ×200).

To analyze the migration of Jurkat cells through the tight junctions (containing claudin-12) of the formed monolayers from the selected adherent cells, we used the same transwell system as described above with small modifications. In order to avoid migration of the adherent cells through the transwell membrane, we used Corning^®^ HTS Transwell^®^ 96 well plates with 3.0 μm pore polycarbonate membrane (cat. No CLS3385, Merck KgaA, Darmstadt, Germany) instead of 8.0 μm. After treatment with monoclonal anti-CLDN12 antibody (blocking the tight junctions) and washing of the cell monolayers formed from A549, LS180, or HeLa cells in the upper chambers, Jurkat cells (1.0 × 10^6^ cells/well) suspended in 150 μL FBS-free medium were added and co-cultured for 24 h at 37 °C in a humidified CO_2_ incubator. A549, LS180, and HeLa cells without anti-CLDN12 antibody treatment served as control groups. Lower chambers were supplemented again with complete medium containing 10% FBS. Migrated Jurkat cells were photographed, counted, and collected for flow cytometric analysis.

### 2.5. Cell Proliferation Assay

An MTT (3-(4,5-dimethylthiazol-2-yl)-2,4-diphenyltetrazolium bromide) assay was used to examine the cell proliferation of the studied cell lines (A549, LS180, and HeLa) after treatment with anti-claudin-12 antibodies or synthetic claudin-12 peptides (p1-p6). Briefly, the cells were seeded (1.0 × 10^5^ cells/well) on 96-well plates (TPP, Trasadingen, Switzerland) and cultured in complete DMEM for 24 h at 37 °C, 5% CO_2_, and high humidity. Then, we added anti-CLDN12 antibody (1 μg/mL) or synthetic peptides (5 μg/mL, p1-p6) representing the extracellular loops of claudin-12, and incubated the cultures for a further 24 h. For the last 3 h of the incubation period (48 h), 10 μL MTT solution (5 mg/mL) was added to each well. Subsequently, the MTT-containing medium was removed, and 100 µL DMSO was added into each well. The cells were incubated for 15 min at room temperature on a shaker in order to dissolve the accumulated formazan crystals. Absorbance was measured at 540 nm using a Synergy-2 reader (BioTek, Winooski, VT, USA). The results were expressed as a percentage of the untreated control (mean ± SE of triplicates, ** *p* < 0.01 vs. the control group).

### 2.6. Flow Cytometric (FACS) Analysis

To investigate whether anti-CLDN12 antibodies can induce apoptotic cell death, we used staining with Annexin V-FITC and propidium iodide (PI) followed by flow cytometric analysis. After the treatment period (24 h) with anti-CLDN12 antibodies, A549, LS180, and HeLa cells were washed twice with FACS buffer (DPBS containing 5% fetal calf serum and 0.05% NaN_3_), harvested by centrifugation at 1500 rpm for 5 min, and resuspended in 500 μL binding buffer. Five microliters of Annexin V-FITC and 5 μL of PI were added to each sample and the cells were incubated for 15 min at room temperature in darkness. Cells were then washed twice and analyzed using a Cytomics FC 500 Flow Cytometer (Beckman Coulter, Indianapolis, IN, USA).

Control Jurkat cells as well as migrated Jurkat cells (after co-culture with the studied adherent cells) were collected and washed with FACS buffer. Then, the cells were stained with FITC-conjugated anti-human CD62L (L-selectin), APC-conjugated anti-human CD11a/CD18 (LFA-1), and PE-conjugated anti-human CD44 antibodies for 30 min at 4 °C in darkness. After that, the cells were washed twice with FACS buffer and subjected to flow cytometry using a Cytomics FC500 instrument (Beckman Coulter, Indianapolis, IN, USA). The levels of the studied biomarkers were compared between the control and migrated Jurkat cells.

### 2.7. Cell Migration Assay with Competitive Inhibition

To evaluate the role of claudin-12 protein in the migration of Jurkat cells through the monolayers from adherent cells (A549, LS180, or HeLa), we again performed the same migration assay described before for migration of Jurkat cells, but without treatment of monolayers with anti-CLDN12 antibody. Here, to the co-culture from Jurkat and adherent cells in the upper chambers of the Transwell^®^ (3.0 μm), we added synthetic peptides (p1, p2, p3, p4, p5, p6) representing parts (loop-1 and loop-2) from the extracellular domains of the protein claudin-12 (Figure 1). Mimicking the extracellular domains of claudin-12 within the tight junctions of the monolayers formed by A549 and LS180 cells, these peptides compete for binding to the migrated Jurkat cells. If our hypothesis for the involvement of tight junction protein claudin-12 in migration during metastasis was true, the number of migrated Jurkat cells through the tight junctions would be reduced by competitive inhibition. Each peptide was tested in triplicate in a final concentration of 5 μg/mL. Co-culture transwells without peptides were used as controls.

### 2.8. Statistical Analysis

All experiments were conducted in triplicate and all data were presented as the mean values ± SE. To compare non-parametric data for statistical significance, the Mann-Whitney *U*-test or Kruskal-Wallis test were applied using the StatView program (SAS Institute). Values of *p* < 0.05 were considered significant (* *p* < 0.05, ** *p* < 0.01, *** *p* < 0.001). All results were compared to those from the controls.

## 3. Results

### 3.1. Expression of Claudin-12

**Figure 2:**
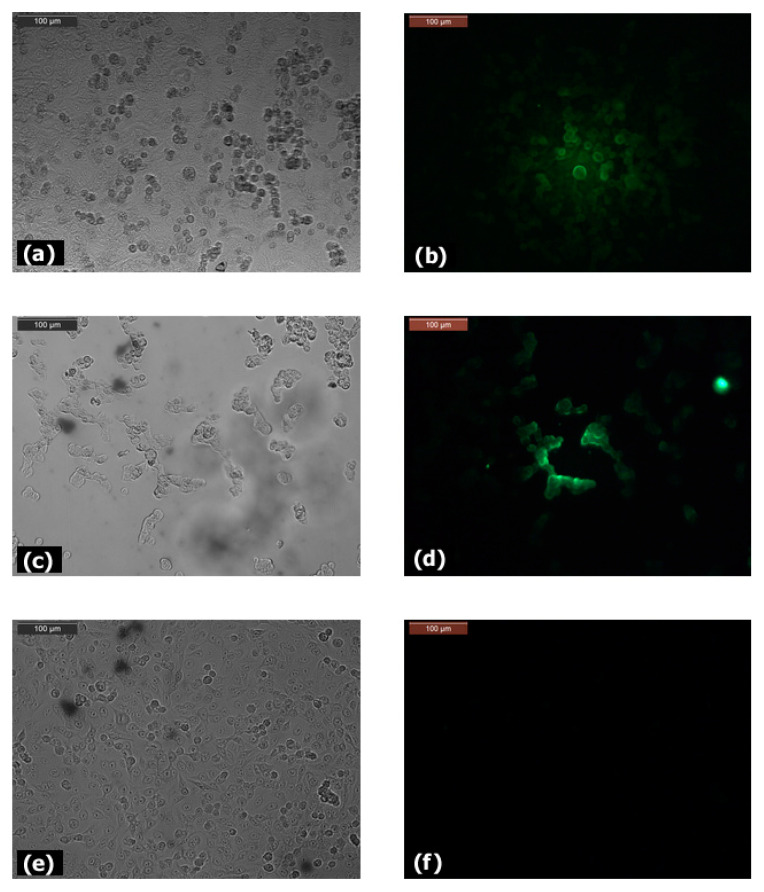
Expression of claudin-12 in A549 (**a**,**b**), LS180 (**c**,**d**), and HeLa (**e**,**f**) cells, cultured for 48 h (**a**,**c**,**e**)—light microscopy; (**b**,**d**,**f**)—fluorescent microscopy). Fixed cells were stained with purified monoclonal anti-CLDN12 antibody and FITC-conjugated secondary anti-mouse IgG. Bar 100 µm.

In order to select a suitable in vitro model for our study, we tested several human cell lines (Caco-2, LS180, HT-29, A549, HeLa, SK-Hep-1) for expression of claudin-12. Taking into account that this protein expressed predominantly in the tight junctions of epithelia, we chose two claudin-12 expressing cell lines (Figure 2a–d) of different origin—A549 (alveolar epithelial cells derived from lung tissue) and LS180 (epithelial cells derived from the colon), and one non-expressing claudin-12 cell line (HeLa) as a negative control (Figure 2e–f). In addition to the selected cell lines, Caco-2 and HT-29 cells also expressed claudin-12, although the signal was weaker, but not the cell line SK-Hep-1, which was claudin-12 negative (data not shown). We decided to use the cell line LS180 instead Caco-2 or HT-29 cells because, in addition, LS180 cells produce mucin.

**Figure 3:**
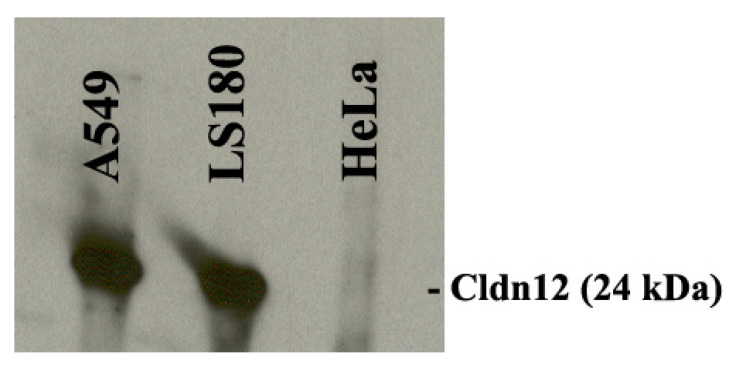
Western blot analysis of claudin-12 protein demonstrating expression in A549 and LS180 cells. HeLa cells displayed no expression of claudin-12.

To confirm our observations from immunofluorescence, we employed Western blotting analysis for the selected cell lines (A549, LS180, and HeLa). Results demonstrated expression of claudin-12 in both A549 and LS180 cell lines, but not in HeLa cells (Figure 3), which corresponded with the data from the immunofluorescence studies.

### 3.2. Anti-Claudin-12 Antibodies Suppress the Migration and Proliferation of Claudin-12 Expressing Cancer Cells, Inducing Apoptosis

**Figure 4:**
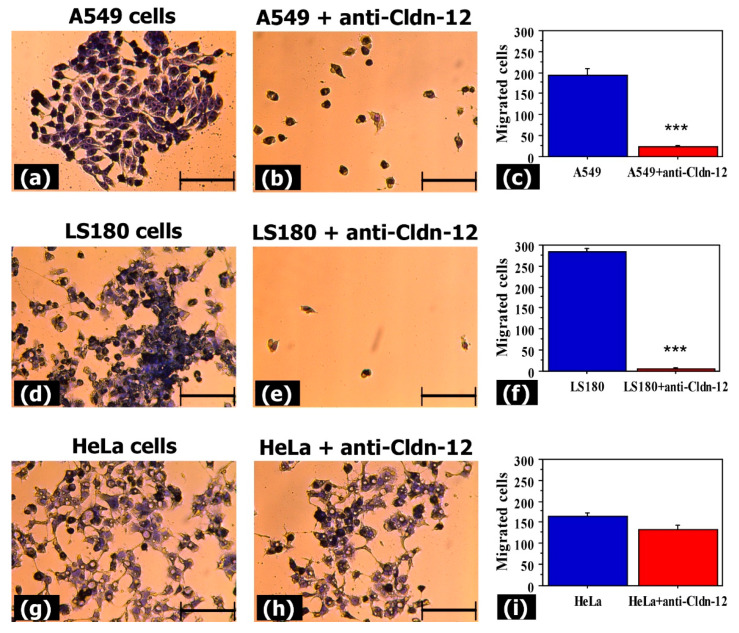
Impact of claudin-12 on the migration abilities of cancer cells expressing claudin-12. A549, LS180, and HeLa cells were treated with anti-CLDN12 antibody, and the Transwell chamber method was used to evaluate the migration of the treated and non-treated cells. Cells were stained with 0.5% crystal violet (**a**,**b**,**d**,**e**,**g**,**h**). Results from the comparative analysis (**c**,**f**,**i**) are presented as mean ± standard error of the mean. *** indicates *p* < 0.001. Bar 100 µm.

To evaluate whether anti-claudin-12 antibodies can influence the migration of claudin-12 expressing cells (A549 and LS180), we used transwell assays. Results showed a significant reduction in the number of migrated A549 and LS180 cells after treatment with anti-claudin-12 antibodies compared with non-treated cells (Figure 4).

The mean numbers of migrated A549 cells with and without antibody treatment were 23 ± 4 and 193 ± 17 cells, respectively (Figure 4a–c). For the LS180 cell line, these values were 6 ± 3 and 283 ± 8 cells, respectively (Figure 4d–f). Our data revealed no significant difference in migration ability between the anti-claudin-12 antibody treated (133 ± 10) and non-treated (165 ± 7) HeLa cells (Figure 4d–f). These analyses suggested that claudin-12 in the tight junctions is involved in the migration of cells expressing this protein.

**Figure 5:**
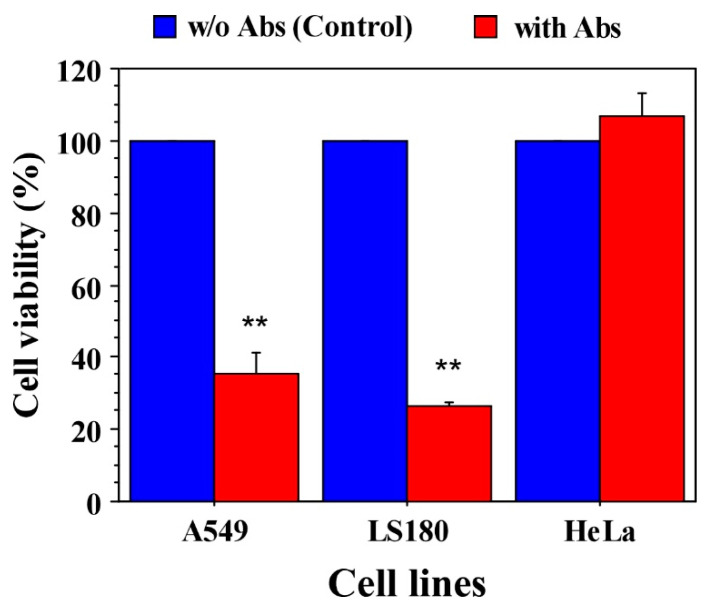
Impact of claudin-12 on the proliferation of cancer cells expressing claudin-12. A549, LS180, and HeLa cells were treated with anti-CLDN12 antibody, and the MTT assay was used to examine the cell proliferation of the treated and non-treated cells. Results are presented as a percentage of viable cells compared to the control (non-treated cells) calculated from triplicates. ** indicates *p* < 0.01.

In addition, to examine the effect of anti-claudin-12 antibodies on cell viability (respectively, cell proliferation), we performed MTT assays using the same strategy of antibody treatment. As shown in Figure 5, antibody treatment resulted in a significant reduction in proliferation of claudin-12 expressing A549 and LS180 cells compared to the controls, and a slight increase in proliferation of HeLa cells. After 48 h, the cell viability of A549 cells decreased to 35.47%, and of LS180 cells, to 26.42% (Figure 5). MTT assays demonstrated that anti-claudin-12 antibodies suppress cell proliferation as well.

**Figure 6:**
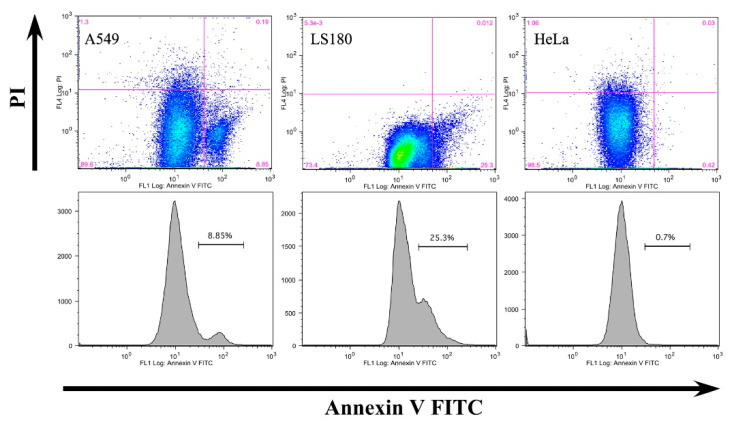
Apoptotic effects of anti-CLDN12 antibodies on A549, LS180, and HeLa cells. The cells were treated with anti-CLDN12 antibodies (1 μg/mL) for 24 h and then stained with annexin V-FITC (horizontal arrow) and propidium iodide (PI, vertical arrow). The percentages of apoptotic cells are shown on the histograms (second row).

To evaluate whether the reduced cell viability is due to apoptosis or necrosis, we conducted flow cytometry. Double staining with Annexin-V FITC and propidium iodide clearly indicated that anti-CLDN12 antibodies induce apoptosis in A549 (8.85%) and LS180 cells (25.3%) (Figure 6), probably via blockade of cell division after binding to claudin-12. HeLa cells were not affected (Figure 6). These data suggest that the inhibition of cell proliferation and migration of claudin-12 expressing cells is due to apoptotic cell death caused by anti-CLDN12 antibodies.

### 3.3. Claudin-12 Is Involved in the Migration of Jurkat Cells through the Tight Junctions

**Figure 7:**
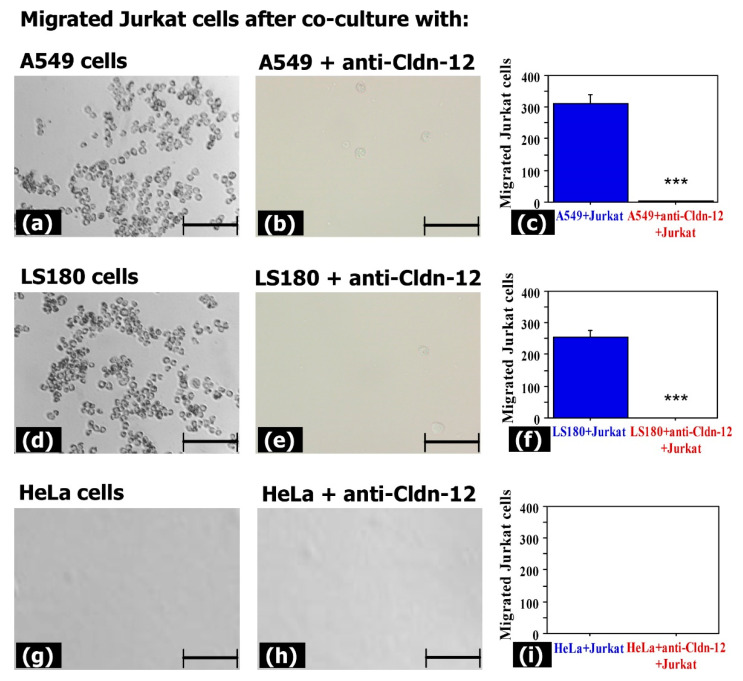
Impact of claudin-12 on the migration abilities of Jurkat cells. Monolayers from A549, LS180, and HeLa cells were treated with anti-CLDN12 antibody and co-cultured with Jurkat cells. The transwell chamber method was used to evaluate the migration of Jurkat cells through the monolayers. Images were taken from the lower chambers where the migrated cells should be (**a**,**b**,**d**,**e**,**g**,**h**). Results from the comparative analysis (**c**,**f**,**i**) are presented as mean ± standard error of the mean. *** indicates *p* < 0.001. Bar 100 µm.

To determine the involvement of claudin-12 in the process of metastasis, we next performed assays similar to the previous transwell assays, but using membranes with 3.0 μm pore size and a co-culture of adherent cells (A549, LS180, or HeLa) and Jurkat cells (Figure 7). The results revealed that Jurkat cells were able to migrate mostly through the tight junctions of the claudin-12 expressing cells (A549 and LS180) that were not pre-treated with anti-claudin-12 antibodies (Figure 7). The mean numbers of Jurkat cells that migrated through A549 and LS180 cells were 310 ± 30 (Figure 7c) and 254 ± 21, respectively (Figure 7f).

We found no differences in the co-culture system HeLa-Jurkat, where migrated Jurkat cells were not detected (Figure 7g–i). These results confirmed that claudin-12 is involved in cell migration during metastasis.

### 3.4. Migrating Jurkat Cells Express Lymphocyte Function-Associated Antigen-1 (LFA-1 Integrin) and L-Selectin (CD62L)

**Figure 8:**
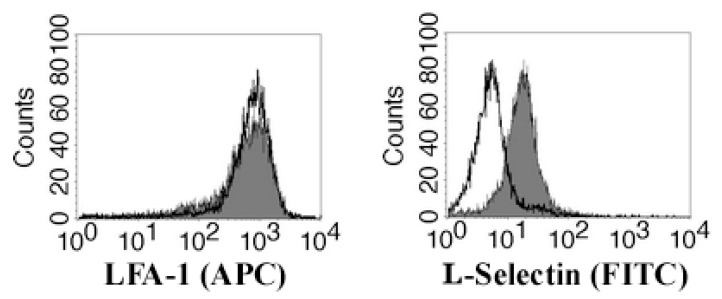
Flow cytometric analysis of migrated Jurkat cells (shaded histograms) and control Jurkat cells (open histograms) for expression of LFA-1 and L-selectin. Cells were stained with anti-human CD11a/CD18 APC-conjugated (for LFA-1) and anti-human CD62L FITC-conjugated (for L-selectin) antibodies. The data are from one representative experiment.

We also performed flow cytometric analysis of the Jurkat cells for expression of LFA-1 and CD62L before co-culturing and after the migration assay. As can be seen in Figure 8, Jurkat cells constitutively express the integrin LFA-1 (before and after migration), while the expression of L-selectin (CD62L) was induced after co-culturing with A549 or LS180 cells. These results suggest that for migration through the tight junctions, Jurkat cells probably use LFA-1 or CD62L, or both molecules. Jurkat cells did not express CD44 glycoprotein (data not shown).

### 3.5. Claudin-12 Peptides Can Block the Migration of Jurkat Cells Through the Tight Junctions

**Figure 9:**
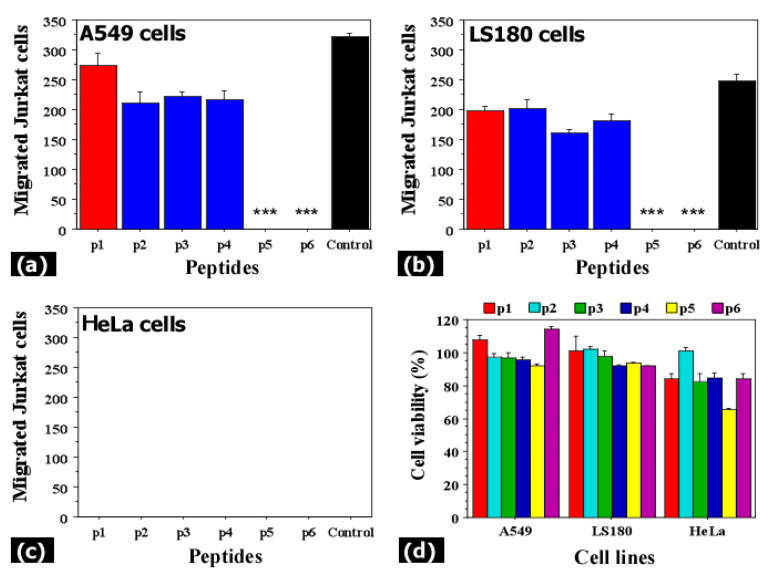
Migration capacity of Jurkat cells after incubation with synthetic claudin-12 peptides (**a**–**c**) and effects of the peptides on A549, LS180, and HeLa cells (**d**). Monolayers from A549 (**a**), LS180 (**b**), and HeLa cells (**c**) were co-cultured for 24 h with Jurkat cells in the presence of 5 μg/mL synthetic claudin-12 peptides. Six synthetic claudin-12 peptides (p1-p6) representing the extracellular domains of the claudin-12 protein were tested as competitors of the claudin-12 in the tight junctions. The transwell chamber method was used to evaluate the migration of Jurkat cells through the monolayers. Results are presented as mean ± standard error of the mean. *** indicates *p* < 0.001. (**d**) A549, LS180, and HeLa cells were treated with 5 μg/mL synthetic claudin-12 peptides (p1–p6) for 24 h, and the MTT assay was used to examine the cell proliferation of the treated and non-treated cells. Results are shown as a percentage of viable cells compared to the control (non-treated cells) calculated from triplicates.

To examine the potential of claudin-12 peptides (derived from the extracellular domains of the claudin-12 protein) to reduce the migration ability of Jurkat cells, we repeated the transwell experiments with co-culture systems, but instead of anti-claudin-12 antibodies, we used synthetic claudin-12 peptides (Figure 9). Our results indicated that two of the used peptides (p5 and p6) completely inhibited the migration of Jurkat cells through the A549 (Figure 9a) and LS180 cells (Figure 9b). Although the other claudin-12 peptides (p1, p2, p3, and p4) slightly reduced the migration of Jurkat cells compared to the control (black bars, without peptides), this inhibition was not significant (Figure 9). Results suggest that Jurkat cells migrate through the tight junctions by binding to claudin-12 and more specifically, to the first extracellular loop (Figure 1), because the peptides p5 and p6 are parts of this domain. The peptide p1 (red bars) that represented the second extracellular loop did not inhibit the migration of Jurkat cells. As shown in Figure 9d, the used synthetic peptides did not have a significant impact on the claudin-12 expressing cells (A549 and LS180). Decreased cell viability (65% from the control) was measured after treatment of HeLa cells with p5. At this point, we do not have an explanation as it was irrelevant to the tasks of this study.

## 4. Discussion

Claudin proteins are integral components of the tight junctions maintaining cell polarity, paracellular permeability, cell proliferation, transformation, and metastasis [21]. It has been shown that during metastasis, expression of certain claudins could increase or decrease in a tissue-specific fashion [17,22,23,24,25,26,27,28,29,30]. The expression and functions of these proteins are regulated by different mechanisms including disintegration of the cell-cell contacts, cytokines, hormones, or other signaling pathways. In our study, we focused on the non-canonical claudin-12 (lacking a PDZ binding domain) and its significance for cell migration during metastasis.

We found that epithelial-derived cancer cell lines from the colon (LS180, Caco-2, HT-29) and the lung (A549) express claudin-12, while liver endothelial SK-Hep-1 and cervix epithelial HeLa cancer cells were claudin-12 negative. This is in agreement with results of other studies that showed expression of claudin-12 in the epithelia and endothelia of the gastrointestinal tract [1] or bronchial epithelial cells [17]. Lack of expression of claudin proteins in HeLa cells (claudin-null cell line) was also previously reported [31,32]. This comparative analysis showed that the cell lines (A549, LS180, HeLa) used as models in this study were correctly selected.

Pretreatment of claudin-12 expressing cells (A549, LS180) with anti-claudin-12 antibodies significantly reduced both migration and proliferation of these cells and induced apoptosis, probably blocking the cytokinesis of cell division after binding to the extracellular domain of claudin-12. Even more, such pretreatment blocked the migration of Jurkat cells through the tight junctions of the formed epithelial cell monolayers during the co-culture. These observations are novel and demonstrate the potential capabilities of anti-claudin-12 antibodies to inhibit the metastatic process in claudin-12 expressing tissues. For example, it has recently been reported that claudin-12 is involved in the epithelial-mesenchymal transition and migration of human bronchial epithelial BEAS-2B cells [17]. In addition, the same researchers found upregulated expression of claudin-12 in lung squamous cell carcinoma (SqCC) tissues, suggesting the CLDN12 gene as a proto-oncogene in SqCC [17]. Similarly, Tian et al. concluded that cytoplasmic overexpression of claudin-12 promotes the proliferation and migration ability of osteosarcoma cells [14]. Taking into account the tissue-specific expression of the claudin proteins, Jiang et al. [21] noted that they could be used as prognostic and diagnostic biomarkers, e.g., claudin-1 for colon cancers, claudin-3 for ovarian cancers, claudin-10 for hepatocellular carcinomas, etc. Thus, claudin-12 may be used as a biomarker for tumor progression and metastasis in the gastrointestinal tract, lung SqCC, and osteosarcomas.

On the other hand, claudin proteins were identified as an attractive target for antitumor therapy [21]. Therefore, blocking expression of claudin-12 with anti-claudin-12 antibodies should have a beneficial effect, inhibiting tumor progression and metastasis in tissues expressing claudin-12. Our results showed that short peptides from the first extracellular loop (Figure 1) of the protein claudin-12 are also able to reduce the migration of Jurkat cells through the tight junctions, suggesting that competitive inhibition mechanisms could be useful in the therapeutic approach applied to cancer metastasis.

We found that migrating Jurkat cells express both integrin LFA-1 and L-selectin (CD62L). It is well known that LFA-1 and L-selectin play a major role in the adhesion of circulating leukocytes to the endothelial cells regulating T cell activation and migration through the endothelium [33,34,35]. Based on our observation that anti-claudin-12 antibodies reduced the number of migrated Jurkat cells, we hypothesized that claudin-12 is a ligand for LFA-1 and/or L-selectin. However, additional studies are needed to prove whether claudin-12 binds to LFA-1 and L-selectin, leading to the disruption of tight junctions and migration of T cells.

## 5. Conclusions

We have shown that A549 and LS180 cells express claudin-12, and that anti-claudin-12 antibodies inhibit cell migration and proliferation and induce apoptosis of claudin-12 expressing cells. Furthermore, synthetic peptides representing the first extracellular loop of claudin-12 reduce the migration of Jurkat cells. Migrating Jurkat cells express LFA-1 and L-selectin. Our findings identified the essential role of claudin-12 in the migration of cancer cells through claudin-12 expressing tissues in the process of metastasis.

## Data Availability

Not applicable.
